# Breeding practices and trait preferences of goat keepers at Lepelle-Nkumpi Local Municipality, South Africa: implication for the design of breeding programmes

**DOI:** 10.1007/s11250-022-03078-x

**Published:** 2022-01-19

**Authors:** Thobela Louis Tyasi, Jones Ng’ambi, Stanley Mogashoa

**Affiliations:** 1 Department of Agricultural Economics and Animal Production, University of Limpopo, Private Bag X1106, Sovenga, 0727 Limpopo South Africa; 2 Department of Agriculture and Animal Health, College of Agriculture and Environmental Sciences, University of South Africa, Pretoria, South Africa

**Keywords:** Breeding practices, Breeding programmes, Selection criteria, Goat keepers, Trait preferences

## Abstract

Identification of breeding practices and trait preferences by livestock keepers for the selection of breeding animals to be parents of the next generations is the crucial step to the successful implementation of community-based breeding program (CBBPs). The study aimed to detect breeding practices and trait preferences by farmers at Lepelle-Nkumpi Local Municipality, South Africa to determine their relevance in establishing a CBBP. A well-structured questionnaire was designed and administered to 183 randomly selected goat keepers from four villages. Chi-square statistics were used to compare categorical variables among villages. Socio-economic factors and reasons for keeping goats were not significantly different (*P* > 0.05) between the four villages. Methods of controlling mating, reasons for not controlling mating, keeping breeding bucks, source of breeding bucks, reasons for culling, and culling methods were significantly different (*P* < 0.05) among villages. The most common trait preferences of goat keepers among the surveyed villages were twinning ability, mothering ability, and body size in breeding does, while in breeding bucks were mating ability, growth rate, and body size. The results from this study are useful for designing CBBPs for goat production in the communal areas of Lepelle-Nkumpi Local Municipality.

## Introduction

South Africa is a comparatively small-scale goat producing country whereby it holds about 1% in the world’s listings of the goat numbers in Africa, it is only 3%, while Eastern Cape has more goats, accounting for 38%, followed by Limpopo with 17%, KwaZulu Natal with 13%, and North West 12% (DAFF 2019). In South Africa, goats are kept by commercial and communal farmers (Slayi et al. 2014; Mdladla et al. 2017). Commercial farmers keep Red Kalahari, Savannah, and Boer goats for meat production, Saanen and Toggenburg goats for milk production, and Angora goats for mohair production (Gwaze et al. 2009). Mostly, communal farmers keep goats to fulfill multiple roles that include manure, traditional ceremonies, skin, milk, meat, and bush encroachment control (Saico and Abul 2007; Gwaze et al. 2010; Chokoe et al. 2020a, b). Over six million goats are raised by communal farmers in South Africa (Chokoe et al. 2020a, b). However, communal goat keepers have low production resulting from limited knowledge in livestock genetic improvements (Yakubu et al. 2019). CBBP is a process of breeding that requires a bottom-up approach where livestock specialists assist farmers to identify and understand their production challenges before designing an improvement program (Nandolo et al. 2016). CBBPs attempt to achieve the genetic improvement of animals through farmer participation practice (Ouedraogo et al. 2020; Zoma-Traore et al. 2021). Knowing traditional farmers’ breeding objectives and trait preferences helps in the development of CBBPs (Berhanu et al. 2012; Fantahun et al. 2016; Yakubu et al. 2020). Breeding objectives aid farmers stick to directions which will attain increased profit (Wurzinger et al. 2011). Therefore, farmers need production improvement, and CBBPs have gained attention as a promising method for the genetic improvement of small ruminants (Manirakiza et al. 2020). Several studies have been conducted on communal goat keepers to identify their breeding objectives, practices, traits and breed preference, and selection criteria to design CBBPs (Meme 2016; Onzima et al. 2018; Abraham et al. 2018; Nguluma et al. 2020; Nguluma et al. 2020; Ramzan et al. 2020). However, based on our knowledge, there are limited studies on breeding objectives and trait preferences of South African goat keepers. Hence, the current study was conducted to investigate the breeding practices and trait preferences of goat keepers with implications for the design of the breeding program.

## Materials and methods

### Description of the study area

The study was conducted in Lepelle-Nkumpi Local Municipality, Capricorn District Municipality of Limpopo province, South Africa. Lepelle-Nkumpi Local Municipality is located at 24° 2585′ S latitude and 29° 6499′ E longitude. The mean annual rainfall is between 453 and 474 mm. The mean annual temperature is approximately 20 °C with an average summer temperature of 23 °C and an average winter temperature of 20 °C. There are very great amounts of livestock species within the Capricorn District, which are goats (44%), followed by cattle (38%), pigs (10%), and sheep (9%). Nearly all the goats in the Capricorn District (98%) are communally farmed. Thus, the existing livestock farming in Lepelle-Nkumpi Local Municipality involves goats, cattle, sheep, and poultry. The vegetation in Lepelle-Nkumpi Municipality is predominantly Savannah Biome (grasses with dispersed trees and shrubs) (Kuyamandi Development Services, 2006).

### Sampling techniques and sample size

The study was conducted following the cross-sectional study design method. A multi-stage sampling procedure was employed whereby Lepelle-Nkumpi Local Municipality was purposively selected as the first stage since the Department of Agriculture, Land Reform, and Rural Development in Limpopo indicated that this local municipality has a higher population of indigenous goats. Four villages were randomly selected, namely Morotse, Sepitsi, Malekapane, and Semiloane. A total 183 goat keepers were randomly selected at Morotse (*n* = 65), Sepitsi (*n* = 51), Malekapane (*n* = 36), and Semiloane (*n* = 31) out of 227 goat keepers (Morotse = 80, Sepitsi = 60, Malekapane = 47, and Semiloane = 40). Lepelle-Nkumpi Local Municipality goat keepers keep their animals in an extensive farming system in the villages.

### Data collection

Data were collected on goat keepers’ breeding knowledge and socioeconomic profile through face-to-face interviews using a semi-structured questionnaire that was designed as described by Haile et al. (2011). The questionnaire was pretested in 5 goat keepers per village to check whether all the questions were adequate, clear, and understandable. The questionnaire was administered to individual household heads responsible for goat farming, but all the members of the household could add any relevant information. Identification of selection criteria for breeding stock, trait, and coat color preferences was done in a participatory manner, as explained by Duguma et al. (2011). Briefly, respondents were provided with the list of ten (10) traits (mating ability, body size, horns, coat color, growth rate, temperament, twinning ability, mothering ability, age at first kidding, and kidding ability) and were asked to choose the traits preferred for the selection of breeding stock. However, the respondents were asked to add any additional traits which were not on the list.

### Data analysis

Data were analyzed using Statistical Package for the Social Sciences version 27 (SPSS, 2020). Chi-square (*χ*^2^) statistics were used to compare categorical variables between four villages. Selection criteria, coat color, and trait preferences were calculated for the importance of each criterion and estimated by computing the index of ranking as explained by Zewdu et al. (2018). Index = sum (3 × rank1 + 2 × rank2 + 1 × rank3) for individual trait/sum (3 × rank1 + 2 × rank2 + 3 × rank1) for overall traits.

## Results

Socio-economic characteristics of respondents (Table 1) and reasons for keeping goats (Table 2) were not significantly different (*P* > 0.05) between the four villages. The information on the number of goats kept by respondents is presented in Table 3. The number of goats kept was more in Morotse, followed by Sepitsi, Semiloane, and Malekapane. Methods of controlling mating, reasons for not controlling mating, keeping breeding bucks, source of breeding bucks, reasons for culling, and culling methods were significantly different (*P* < 0.05) between the four villages (Table 4). The index was used for computing the importance of the traits. The findings showed that overall, body size (0.329), mating ability (0.305), growth rate (0.228), temperament (0.037), coat color (0.082), and horns (0.019) were indicated as the important traits for the selection of breeding bucks (Table 5) in all villages. Twinning ability (0.328), body size (0.325), mothering ability (0.141), temperament (0.065), age at first kidding (0.051), kidding ability (0.051), and coat color (0.039) were recognized as the important traits in overall for selection of the breeding does (Table 6) in all the villages.

**Table 1:** Socio-economic characteristics of the respondents

Factor	Villages					
	Morotse no. (%)	Sepitsi no. (%)	Malekapane no. (%)	Semiloane no. (%)	Chi-square	*P*-value
Sex						
Male	38 (58)	28 (55)	21 (58)	19 (61)		
Female	27 (42)	23 (42)	15 (42)	12 (39)	0.35	0.95^ ns^
Age						
< 50 yrs	20 (31)	14 (28)	10 (28)	9 (29)		
50–70 yrs	28 (43)	24 (48)	17 (47)	14 (45)		
> 70 yrs	17 (26)	13 (24)	9 (25)	8 (26)	0.28	1.00^ ns^
Educational level						
No formal	3 (5)	2 (4)	2 (6)	2 (6)		
Primary	0 (0)	0 (0)	0 (0)	1 (3)		
Sec. & abv	62 (95)	49 (96)	34 (94)	28 (90)	5.27	0.51^ ns^
Marital status						
Single	3 (5)	2 (4)	1 (3)	1 (3)		
Married	61 (94)	47 (92)	35 (97)	30 (97)		
Widow	1 (2)	2 (4)	0 (0)	0 (0)	3.04	0.80^ ns^

*Yrs*, years; *Sec & abv*, secondary and above; ^*ns*^, not significant

**Table 2:** Proportions of respondents keeping goats for particular reasons across villages

Reasons	Villages					
	Morotse no. (%)	Sepitsi no. (%)	Malekapane no. (%)	Semiloane no. (%)	Chi-sqaure	*P*-value
Companionship	2 (6)	4 (6)	0 (0)	5 (10)		
Meat	9 (25)	12 (18)	9 (29)	11 (22)		
Milk	4 (11)	5 (8)	3 (10)	5 (10)		
Status	6 (17)	3 (5)	2 (6)	5 (10)		
Tradition	8 (22)	19 (29)	9 (29)	9 (18)		
Sale	2 (6)	14 (22)	6 (19)	7 (14)		
Security	5 (14)	8 (12)	2 (6)	9 (18)	16.63	0.55^ ns^

^*ns*^, not significant

**Table 3:** Percentages and numbers of respondents indicating goat herd size

Community	No. of goats	Mean	Min	Max	< 21 goats	21–40 goats	> 40 goats
Morotse	2413	37.1	10	63	20.00%	36.92%	43.08%
Sepitsi	1714	34.3	9	70	35.30%	27.45%	37.25%
Malekapane	1059	29.4	10	55	33.33%	55.56%	11.11%
Semiloane	1064	33.6	10	62	25.80%	41.94%	32.26%

**Table 4:** Proportions of goat keepers for breeding practices

Breeding practice	Villages					
	Morotse no. (%)	Sepitsi no. (%)	Malekapane no. (%)	Semiloane no. (%)	Chi-square	*P-*value
Breeding season						
Spring	50 (76.92)	39 (76.47)	31 (86.11)	27 (87.10)		
Autumn	15 (23.08)	12 (23.53)	5 (13.89)	4 (12.90)	2.62	0.45^ ns^
Mating methods						
Natural	65 (100.00)	51 (100.00)	36 (100.00)	31 (100.00)		
Artificial insemination (AI)	0 (0.00)	0 (0.00)	0 (0.00)	0 (0.00)		
Controlled mating						
Yes	31 (47.69)	32 (62.75)	19 (52.78)	18 (58.06)		
No	34 (52.31)	19 (37.25)	17 (47.22)	13 (41.94)	2.81	0.42^ ns^
Methods of controlling mating						
Castration	20 (30.77)	25 (49.02)	12 (33.33)	12 (38.71)		
Culling	20 (30.77)	11 (21.57)	18 (50.00)	13 (41.94)		
Castration and culling	25 (38.46)	15 (29.41)	6 (16.67)	6 (19.35)	13.54	0.04*
Reasons for not control mating						
Goats grazing together	27 (41.54)	11 (21.57)	16 (44.44)	6 (51.61)		
Lack of awareness	13 (20.00)	24 (47.06)	9 (25.00)	16 (19.35		
Both	25 (38.46)	16 (31.37)	11 (30.56)	9 (29.03)	15.43	0.02*
Keep breeding bucks						
Yes	34 (52.31)	18 (35.29)	21 (58.33)	7 (22.58)		
No	31 (47.69)	33 (64.71)	15 (41.67)	24 (77.42)	12.17	0.01**
Reasons for keeping bucks						
Mating	31 (47.69)	30 (58.82)	17 (47.22)	21 (67.74)		
Fattening	18 (27.69)	15 (29.41)	13 (36.11)	7 (22.58)		
Mating and fattening	16 (24.62)	6 (11.76)	6 (16.67)	3 (9.68)	7.23	0.30^ ns^
Breeds of breeding bucks						
Indigenous	45 (69.23)	30 (58.82)	26 (72.22)	27 (87.10)		
Exotic	20 (30.77)	21 (41.18)	10 (27.78)	4 (12.90)	7.44	0.06^ ns^
Source of breeding bucks						
Own	21 (32.31)	20 (39.22)	11 (30.56)	9 (29.03)		
Community	18 (27.69)	25 (49.02)	16 (44.44)	17 (54.84)		
Purchase	26 (40.00)	6 (11.76)	9 (25.00)	5 (16.13)	16.23	0.01*
Reasons for culling						
Poor reproduction	17 (26.15)	4 (7.84)	18 (50.00)	12 (38.71)		
Undesired body confirmation	5 (7.69)	6 (11.76)	10 (27.78)	2 (6.45)		
Diseases	6 (9.23)	3 (5.88)	3 (8.33)	0 (0.00)		
Old age	21 (32.31)	22 (43.14)	5 (13.89)	10 (32.26)		
Undesired coat color	16(24.62)	16 (31.37)	0 (0.00)	7 (22.58)	42.97	< 0.001*
Culling method						
Selling	21 (32.31)	6 (11.76)	6 (16.67)	7(22.58)		
Slaughtering	38 (58.46)	37 (72.55)	28(77.78)	21(67.74)		
Exchange	6 (9.23)	8 (15.69)	2(5.56)	3(9.68)	29.45	< 0.001*

^*^, significant; ^*ns*^, not significant

**Table 5:** Ranks and indices for trait preference in breeding bucks

Trait	Morotse (*n* = 65)				Sepitsi (*n* = 51)				Malekapane (*n* = 36)				Semiloane (*n* = 31)				
	R1	R2	R3	Index	R1	R2	R3	Index	R1	R2	R3	Index	R1	R2	R3	Index	Overall index
Mating ability	20	25	10	0.308	16	20	12	0.327	11	12	10	0.310	9	10	4	0.274	0.305
Body size	25	12	20	0.305	20	12	13	0.317	16	14	9	0.394	11	8	7	0.301	0.329
Horns	0	1	9	0.028	0	0	5	0.016	0	0	0	0.000	0	3	0	0.032	0.019
Coat color	0	7	6	0.051	0	7	10	0.078	1	4	9	0.093	2	2	10	0.108	0.082
Growth rate	17	15	8	0.228	14	12	9	0.245	8	6	4	0.185	9	6	8	0.253	0.228
Temperament	3	5	12	0.079	1	0	2	0.016	0	0	4	0.019	0	2	2	0.032	0.037

*R1–R3*, rank 1 to rank 3; *n*, sample size

**Table 6:** Ranks and indices for trait preference in breeding does

Traits	Morotse (*n* = 65)				Sepitsi (*n* = 51)				Malekapane (*n* = 36)				Semiloane (*n* = 31)				
	R1	R2	R3	Index	R1	R2	R3	Index	R1	R2	R3	Index	R1	R2	R3	Index	Overall index
Twinning ability	23	19	17	0.303	20	17	14	0.333	14	13	10	0.343	12	10	9	0.333	0.328
Body size	19	23	12	0.277	13	19	16	0.314	12	16	9	0.343	13	11	10	0.366	0.325
Mothering ability	7	9	14	0.154	8	10	9	0.176	5	4	8	0.157	2	4	1	0.075	0.141
Temperament	3	2	9	0.072	4	0	8	0.078	0	0	5	0.046	0	1	5	0.065	0.065
Age at 1st kidding	4	1	10	0.077	2	3	1	0.039	2	2	1	0.046	2	0	2	0.043	0.051
Coat color	3	5	0	0.041	1	1	0	0.013	1	0	3	0.037	2	1	3	0.065	0.039
Kidding ability	6	6	3	0.077	3	1	3	0.046	2	1	0	0.028	0	4	1	0.054	0.051

*R1–R3*, rank 1 to rank 3; *n*, sample

## Discussion

Identification of breeding practices and traits preferred by communal livestock keepers is an important step to the successful implementation of a suitable breeding program (Ouedraogo et al. 2020). Socio-economic characteristics of the surveyed Lepelle-Nkumpi Local Municipality goat keepers have been documented in this study. The findings indicated that there were no significant differences observed in socio-economic factors of goat keepers between the four villages. Men were the majority of goat keepers in the current study. However, the men were the majority of goat keepers, and this was expected due to traditional and cultural customary patterns of South African rural people who consider man is the head of the household and likely to have a final say in issues related to the keeping of livestock. The characteristics attached to goats’ ownership in the current study are comparable with the findings of Onzima et al. (2018) when they revealed that the majority (84.8%) of farmers who kept goats were males. Sheriff et al. (2020) also observed that most (67.5%) of farmers who kept goats, especially indigenous breeds were males. The distribution of ownership of livestock species between sexes (men and women) influences the type of livestock raised by the community (Bravo-Baumann 2000). For instance, cattle, sheep, goats, donkeys, and horses are commonly owned by males while pigs and poultry are commonly owned by females (Sheriff et al. 2020). Most of the goat keepers interviewed in the current study had secondary and above levels of education, which suggests that goat keeping activity was mostly practiced by people who can read and write. Thus, it might be easy to train them to practice new approaches such as CBBPs for improved and profitable goat production. This remark disagrees with the result of Mtshali et al. (2021) which showed that most (77.1%) of goat keepers in the North West province of South Africa had primary education, while those with secondary education levels amounted to 45.7 and 2.9% were those with tertiary education level. The present study revealed that there is a variation in the average number of goats kept per household at Morotse (23.12), Sepitsi (34.32), Malekapane (29.42), and Semiloane (33.61). These findings are comparable with studies that reported large herd sizes (Abegaz et al. 2013; Fantahum et al. 2016; Sheriff et al. 2020). Biruh et al. (2017) recorded the largest herd size (54.7) at Benatsemay. On the other hand, small herd sizes (6.10 and 4.55) were reported by Fantahum et al. (2016). Previous research findings from South Africa revealed that the average number of indigenous goats kept by respondents in the North West province was 19.9 (Mdladla et al. 2017) and 21 (Mtshali et al. 2021), and in KwaZulu-Natal, it was 17.4 (Mahlobo 2016). A small number of animals per household could be problematic for selection during breeding and might increase inbreeding and reduce genetic gain (Abebe et al. 2020). Therefore, the goat keepers with a small number of goats must cooperate and take decisions as a group of goat keepers and exchange breeding bucks to improve the performance of their animals. Although goats played a multipurpose role across sampled villages as meat providers, means of performing customary activities, sales of meat were considered the overriding goal of keeping goats. The present finding is consistent with other studies conducted in the developing countries that underscored the importance of goats in generating income (Lorato et al. 2015; Fantahun et al. 2016; Lorato 2016; Onzima et al. 2018; Onzima et al. 2018; Sheriff et al. 2020; Nguluma et al. 2020). According to Mtshali et al. (2021), goat keepers in South Africa sell their goats to generate income. Goat keepers in the present study did not consider milk production as the primary purpose of raising goats. This outcome is in disagreement with previous studies (Kosgey et al. 2008; Hassen and Tesfaye 2014; Abraham et al. 2018). This might be due to cultural differences. However, the findings of this study agree with Dubeuf (2010), Legese et al. (2014), Dossa et al. (2015). The other reason behind the low percentage of milk production in the present study might be a lack of awareness regarding the nutritional importance of goat milk. Therefore, considering the reasons behind the keeping of goats in smallholder farming is vital when a designing breeding program. The importance of farmers’ attributes to multipurpose use of goats recommends that CBBPs might have a good probability of achievement in the studied villages. Valuing indigenous information is also important to guarantee a sustainable breeding program at the village level (Abebe et al. 2020). There were statistically significant differences observed on methods of controlling mating, reasons for not controlling mating, keeping breeding bucks, source of breeding bucks, reasons for culling and culling methods between villages. As expected, the majority of goat keepers in the studied villages practiced natural mating system, and this might be due to a lack of resources and knowledge in practicing the artificial insemination method. The majority of farmers (52.31%; 47.22%, respectively) who did not control herd mating is because their goats shared grazing in communal land. This result is consistent with the findings of (Lorato 2016), who stated that the majority of goat keepers in Ethiopia failed to control mating as a result of constraints imposed by the shared grazing system. Nonetheless, the present study is in disagreement with the report of Nguluma et al. (2020), which indicates that the majority of goat keepers in Tanzania controlled mating using the apron technique in males. Nguluma et al. (2020) further emphasized that it is possible to control herd mating in communal farming using the apron technique in males. The apron technique was reported by Peacock (1996) as an effective traditional way of controlling mating by trapping it around the buck’s waist, which then blocks him from being able to breed with female goats until the farmer decides to remove it to allow breeding. According to Nguluma et al. (2020), the effectiveness of the apron technique might be impractical for farmers who are occupied by multiple functions since it needs to be frequently checked. However, some of farmers in the study villages (Morotse = 20%; Sepitsi = 25%; Malekapane = 12%; Semiloane = 12%) controlled herd mating through castration. Castration is a better practice which increases fat deposition (Kebede et al. 2008). Therefore, farmers in the current study might practice castration since it is a better excise that can positively control mating and prohibits inbreeding. The castration method helps to improve meat quality by removing the smell of the meat **(**Gkarane et al. 2017). Males not selected for breeding must be castrated through the consensus of the community members sharing the grazing sites to implement successful CBBPs, according to Haile et al. (2011). Controlled mating is vital for the genetic improvement of animals as it enables farmers to avoid non-selective mating and inbreeding. The advantage of controlled mating is that only selected animals can pass on their genes to the next herd generation for genetic improvement. The majority of goat keepers relied on bucks from other herds for breeding theirs. This result is similar to the findings of Meme (2016), who found that majority of goat keepers were depending on the community bucks for breeding in Ethiopia. For this reason, the implementation of a breeding program in the studied villages needs to consider the involvement of all goat keepers within each community. Participation of farmers in evaluating breeding practices is vital if a livestock improvement program is to be attained (Mueller et al. 2015). Onzima et al. (2018) reported that the use of preferences based on farmers’ weightings on traits has become a powerful tool for livestock farmers then ranking their animals. Several studies in Africa have used the participatory method to disseminate information for the implementation of CBBPs for goat keepers (Bett et al. 2009; Gebreyesus et al. 2013; Fantahun et al. 2016; Lorato 2016; Meme 2016; Lorato et al. 2017; Onzima et al. 2018; Ramzan et al. 2020; Sheriff et al. 2020). Across all four villages in the present study, body size, mothering ability, and twinning ability were considered very important for breeding does while mating ability, body size, and growth rate for breeding bucks were considered very important by goat keepers. Body size has severally been reported as the most preferred trait in breeding does and bucks by farmers in Ethiopia (Meme 2016; Abraham et al. 2018; Sheriff et al. 2020). In a recent study with indigenous goats in South Africa, goat keepers similarly preferred body size and growth rate for breeding does and bucks (Mtshali et al. 2021). Our findings on trait preferences of breeding bucks do emphasize the importance of body size and growth rate. This is because body size attracts high selling prices while fast-growing animals achieve market weight quickly. However, increasing body size in animals showed a reduction in production efficiency due to higher maintenance costs and increased mortality (Farias et al. 2018). Twinning ability was ranked as an important trait in the selection of breeding does (Lorato et al. 2015; Fantahun et al. 2016; Sheriff et al. 2020). These results are contrary to the report of Nguluma et al. (2020), who found that coat color was ranked as an important trait for the choices of breeding bucks and does. Therefore, coat color also needs to be considered for a sustainable breeding program since goat keepers prefer specific coat colors for traditional purposes. Therefore, CBBPs in the study villages should focus on body size, mothering ability, twinning ability, growth rate, and mating ability. In conclusion, variations were observed in breeding practices (methods of controlling mating, reasons for not controlling mating, keeping breeding bucks, source of breeding bucks, reasons for culling, and culling methods) between the four villages. The study revealed that goat keepers had their highest preference for twinning ability, body size, and good mothering ability in breeding does and good mating ability, body size, and growth rate in breeding bucks. The participatory approach employed in the current study might be useful in identifying breeding practices and farmers’ traits of preferences for a sustainable breed improvement program of goats in communal farming. Breeding practices and trait preferences identified in the current study need to be considered in designing and implementing of CBBPs in the studied villages.

## Data Availability

The data presented in the current study are available on request from the corresponding author.
